# Different infant formulas can activate toll-like receptor 9 in vitro and inhibit interleukin 6 in human primary intestinal epithelial cells

**DOI:** 10.1007/s00394-024-03507-7

**Published:** 2024-11-21

**Authors:** Kathrin Hedegger, Theresa Hommel, Monika Schaubeck, Martina Gimpfl, Maik Dahlhoff

**Affiliations:** 1 Institute of Molecular Animal Breeding and Biotechnology, Gene Center, LMU München, Feodor-Lynen-Straße 25, 81377 Munich, Germany; 2 Institute of in vivo and in vitro Models, University of Veterinary Medicine Vienna, Veterinärplatz 1, 1210 Vienna, Austria; 3 HiPP GmbH & Co. Vertrieb KG, Georg-Hipp-Straße 7, 85276 Pfaffenhofen, Germany

**Keywords:** TLR4, TLR9, Infant formula, Intestine, IL6, NEC

## Abstract

**Purpose:**

Necrotizing enterocolitis (NEC) is the most severe gastrointestinal disease in preterm infants caused by an exaggerated intestinal epithelial immune activation. Several studies show that modulation of toll-like receptor 9 (TLR9) activity may have positive effects on preventing intestinal inflammatory mechanisms ultimately resulting in NEC development. In this study, the effect of various infant formulas (IF) and the probiotic strain *Limosilactobacillus fermentum* CECT5716 on TLR9 activation were analyzed in vitro.

**Methods:**

First, TLR4 and TLR9 expression was analyzed on human primary intestinal epithelial cells (P-IECs) by qPCR and Western blot analysis. Then genetically designed HEK-Dual™ hTLR9 (NF/IL8) reporter cells (HEK-Dual) were treated with different IFs, *L. fermentum* CECT5716, and different functional components to measure TLR9 activation via luminescence. Finally, the IFs were investigated in P-IECs to analyze TLR downstream signaling by Western blot analysis.

**Results:**

IFs containing intact protein and *L. fermentum* CECT5716 activated TLR9 in HEK-Dual cells, but the functional components lactoferrin, L-5-methyltetrahydrofolate, and hydrolyzed whey proteins failed to activate TLR9. In P-IECs, the IFs induced increased phosphorylation of MAPK8/9 of the TLR signaling pathway and significantly reduced IL6 levels. Consistently, IL6 levels were increased in P-IECs when TLR9-signaling was inhibited. Interestingly, *L. fermentum* CECT5716 enhanced TLR9-signaling and increased NF-kappa-B inhibitor alpha-phosphorylation.

**Conclusion:**

We found out that the used control formula, prebiotic formula, prebiotic formula with hydrolyzed-protein, and *L. fermentum* CECT5716 reduce IL6 levels in human P-IECs through TLR9 activation. *L. fermentum* CECT5716 and the here tested IFs could be a promising approach for modulation of gut health in infants.

## Introduction

Necrotizing enterocolitis (NEC) is the most common gastrointestinal disease with a still unknown cause and mechanism, affecting the mortality in preterm infants with very low birth weight [1]. NEC has a mortality rate of 23.5% and is a result of inflammation and necrosis. It affects the small and large intestine, and is often associated with sepsis and peritonitis [2]. As soon as enteral feeding is possible, fortified maternal milk is the best protection against NEC. If maternal or donor milk is not available, preterm formula, which is adapted to the special needs of this age group, provides a suitable source of nutrition. Therefore, ideally composed preterm formula contains ingredients which support healthy gut and tissue maturation according to the special requirements of premature infants, as lactoferrin and milk oligosaccharides [3, 4]. The ingredients should stimulate the innate immune system in order to support an anti-inflammatory intestinal milieu, e.g. by stimulation of anti-inflammatory mechanisms. One of these protective mechanisms might be mediated by the stimulation of toll-like receptor (TLR) signaling through extracellular vesicles or expressed TLRs in cells of human milk [5, 6]. TLRs play a key role in the innate immune system [7] and are expressed in different immune cells such as lymphocytes, natural killer cells, macrophages, and dendritic cells, but they are also expressed in keratinocytes and intestinal epithelial cells [8–10]. The TLR family consists of 13 members performing essential and different roles in innate immune system signaling. Therefore, different strategies have been developed to prevent NEC development by modulating TLR signaling. Independent studies demonstrated upregulated TLR4 signaling, with downregulated TLR9 signaling during NEC and other gastrointestinal inflammatory diseases, indicating TLR4 and TLR9 to act antagonistic in NEC development [11–13]. TLR4 is activated by lipopolysaccharides (LPS) and bacterial heat shock proteins and has been described of being involved in intestinal barrier failure suggesting an important role in the pathogenesis of NEC [14, 15]. TLR4 inhibition by components isolated from human milk resulted in protection against NEC in a preclinical murine model. Good et al. 2015 showed that breast milk protected mice against NEC by inhibiting TLR4, which was shown to be mediated by epidermal growth factor receptor (EGFR) [16]. TLR4 is one of the best studied TLRs in human intestinal epithelial cells, but little is known about the function of TLR9 in the gastrointestinal tract. TLR9 is activated by unmethylated cytosine phosphate-guanosine (CpG) oligodeoxynucleotides and its dysregulation results in inflammatory diseases driven by interferon gamma [17, 18]. After ligand binding, both receptor subunits are bound by myeloid differentiation primary response gene 88 (MyD88) followed by interleukin receptor-associated kinase 4 (IRAK4), IRAK1, IRAK2, and IRAK3 [7]. Downstream of the IRAK kinases, the mitogen-activated protein kinase (MAPK) family is phosphorylated to activate different transcription factors, such as nuclear factor kappa B (NFKB).

Probiotic and postbiotic compounds were reported to have a protective effect against NEC by attenuating TLR4 signaling or through TLR9 activation. In a preclinical model of NEC in preterm piglets, *Lacticaseibacillus rhamnosus* HN001 reduced intestinal inflammation via activation of TLR9 [19]. Furthermore, the TLR4 signaling was reduced by *L. rhamnosus* HN001 as shown by Good et al. 2014 [19]. Therefore, the authors concluded that* L. rhamnosus* HN001 could be a suitable candidate for preventive strategies for infants at risk of NEC. In conclusion, these findings suggest that TLR9 activation perhaps through infant formulas (IFs) could help to improve the intestinal barrier of preterm infants in order to avoid, respectively reduce inflammatory intestinal diseases.

## Materials

### Infant formulas and functional compounds

Test formulas (Fig. 2g) were either ready-to-use or prepared according to the manufacturer’s instructions and were diluted with cell culture medium to the indicated concentrations. The used IFs were characterized by varied combinations of prebiotics (galacto-oligosaccharides, GOS) and/or probiotics (*L. fermentum* CECT5716) and by different protein structure (intact vs. extensively hydrolyzed).

We used a control formula without pre- and probiotics but with intact protein (CF), an infant formula with prebiotics and intact protein (PF), an infant formula with prebiotics and extensively hydrolyzed whey protein (PHF), an infant formula with locust bean gum, probiotics and intact protein (LBGF), and an infant formula low in lactose with pro- and prebiotics, beta-palmitate and extensively hydrolyzed whey protein (RLF) (Fig. 2g). These formulas were isocaloric (66 kcal/100 ml), had an identical fat content (3.6 g/100 ml) and comparable amounts of carbohydrates (6.9–7.1 g/100 ml) and protein (1.25–1.3 g/100 ml). All IFs used in the study were provided by HiPP GmbH & Co. Vertrieb KG, Pfaffenhofen, Germany and comply with all relevant legal requirements current at that time.

In order to test the distinct effects of functional compounds, we used extensively hydrolyzed whey protein (HWP; Peptigen® IF-3080, supplied by Arla Foods Ingredients, Videbæk, Denmark), Lactoferrin (LF; supplied by Milei, Leutkirch, Germany) and L-5-methyltetrahydrofolate (L-5-MTHF; supplied by Merck & Cie, Schaffhausen, Switzerland).

### Cell culture and stimulation experiments

To measure the TLR9 activity the HEK-Dual™ hTLR9 (NF/IL8) reporter cells (HEK-Dual cells) were purchased from InvivoGen (Toulouse, France). Cells were stably transfected with the human *TLR9* gene and with two reporter genes: secreted embryonic alkaline phosphatase (SEAP) and Lucia luciferase, which are inducible by NFKB/AP-1 or interleukin 8 (IL8), respectively. Due to a triple knockout of endogenous TLR3, TLR5, and tumor necrosis factor receptor (TNFR), an unspecific TLR activation is avoided. TLR9 activation induces the expression of NFKB/AP1 and consequently of the SEAP reporter. Additionally, Lucia luciferase is placed under the IL8 promoter and is expressed upon TLR9 activation. Both reporter proteins can be read-out photometrically or by luminescence, respectively, after QUANTI-Blue™ or QUANTI-Luc™ treatment, respectively. Cells were maintained in high-glucose Dulbecco’s Modified Eagle Medium (DMEM, Sigma, Darmstadt, Germany), supplemented with 2 mM L-glutamine, 10% (v/v) heat-inactivated fetal bovine serum (FBS, Sigma), 100 U/ml penicillin, 100 μg/ml streptomycin, 100 μg/ml Normocin, 100 µg/ml Hygromycin, 50 µg/ml Zeocin at 37 °C and 5% CO_2_. Mycoplasma testing was done for all cultured cells, using a mycoplasma detection kit (PlasmoTest, InvivoGen). Cells were used for experiments at up to a maximum of 15 passages to avoid genotypic changes. At a confluence of 80% to 90%, cells were passaged or plated for experiments in 96 well plates. TrypLE Express (Thermo Fisher Scientific, Waltham, MA, USA) was used for cell detachment. 50 µl QUANTI-Luc™ solution (InvivoGen) was added to 20 µl supernatant of HEK-Dual cell and the luminescence was measured and quantified using the Infinite M1000 microplate reader (TECAN, Männedorf, Switzerland) according to the manufacturer`s instructions. Readouts were indicated in relative light units (RLUs).

In all experiments one vial with HEK-Dual cells was treated for 24 h with 0.1 µg/mL TLR9 agonist, ODN2006 (InvivoGen), as a positive control. To inhibit TLR9, cells were pre-treated with 0.1 µg/mL INH-18 (InvivoGen) and 0.1 µg/mL ODN2006 for 24 h. Infant formula was prepared according to the manufacturer’s instructions or provided ready-to-use and diluted with DMEM to the indicated concentrations 1:25 or 1:100. *L. fermentum* CECT5716 was diluted to 1 × 10^8^, 1 × 10^9^, 1 × 10^10^, and 1 × 10^11^ colony-forming units (CFUs) per mL and then diluted with DMEM to the indicated formula concentrations of 1:25 or 1:100 before being applied to the cells. For heat inactivation, bacteria were heated at 90°C for two hours. For lysis, bacteria were sonicated (ultrasound device UW200, BANDELIN electronic, Berlin, Germany) on ice with 60% power at 0.5 s. intervals for 5 min. Afterwards, lysates were centrifuged for 20 min at 4220 x*g* and 4 °C, and supernatant was obtained. The cells were incubated for 24 h with 1/100 diluted IF or 0.1 µg/mL TLR9 agonist (ODN2006) and compared to non-treated basal conditions (medium only). 50 µl QUANTI-Luc™ solution (InvivoGen) was added to 20 µl supernatant of HEK-Dual cell and the luminescence was measured in a TECAN infinite M1000 (TECAN, Männedorf, Switzerland) according to the manufacturer`s instructions.

### MTT cell viability assay

Cell viability was assessed by measuring 3-(4,5-dimethylthiazol-2-yl)-2,5-diphenyltetrazolium bromide (MTT) with a colorimetric assay, as MTT is reduced to purple formazan in living cells (Thermo Fisher Scientific). 1500 cells were plated into each well of a 96 well plate and cultivated in various dilutions (1/10–1/1000) of IF with DMEM medium for 48 h until they became 90% confluent. Afterwards, the cells were incubated in 100 µL MTT solution for 2 h at 37 °C. For the MTT colorimetric assay, we followed the instructions of the manufacturer. Dilutions of 1/25 and higher were found to be non-toxic to cells.

### Human primary intestinal epithelial cell experiments

Adult human primary intestinal epithelial cells (P-IECs) were purchased from PELOBiotech GmbH (Martinsried, Germany). The cells were maintained in complete epithelial growth medium (PELOBiotech GmbH), supplemented with 0.1% insulin-transferrin-selenium, 0.1% epidermal growth factor, 0.1% hydrocortisone, 1% L-glutamine, 1% antibiotic–antimycotic solution and 5% FBS provided by PELOBiotech and according to the manufacturer’s instructions at 37 °C and 5% CO_2_. Only cells of the passages 3 to 6 were used for experiments. For stimulation experiments, the cells were plated in 10 cm dishes, grown to a confluence of 90%, and incubated with 2.5 µM TLR4 inhibitor CLI-095 (InvivoGen) or with 2.5 µM TLR9 antagonist INH-18 over night. Stimulation was then performed using 2.5 µM ODN2006 and 50 ng/mL of the TLR4 agonist lipopolysaccharide (LPS, Sigma), respectively for 30 min. For further experiments, IF and human milk were diluted 1/50 with medium and added to the cells for 30 min. Cells were then washed twice with sterile, ice-cold phosphate-buffered saline (PBS) and either used for protein analysis or resuspended in TRIzol® (Invitrogen, Darmstadt, Germany) for RNA extraction.

### Western blot analysis

As previously described [20], cells were lysed in a TRIS-based buffer (50 mM Tris, 150 mM NaCl, 1% NP40, 10% glycerol, 1M EDTA; freshly supplemented with protease and phosphatase inhibitors (Roche, Mannheim, Germany)) and total protein was applied to Western blot analysis, allowing for the detection of phosphorylation and total expression of intracellular TLR signaling proteins. Equal concentrations of total protein were electrophoresed on 10% polyacrylamide-sodium dodecyl sulfate gels and blotted to PVDF-membranes (GE Healthcare, Munich, Germany). The membranes were blocked with 5% milk and incubated with the primary antibodies overnight at 4 °C and washed. The following primary antibodies were used: rabbit polyclonal anti-phospho-MAPK1/2 (Thr202/Tyr204; 1:2000; #9101; Cell Signaling, Ipswich, USA), rabbit monoclonal anti-phospho-IKBA (Ser32; 1:2000; #2859; Cell Signaling), rabbit monoclonal anti-phospho-MAPK14 (Thr180/Tyr182; 1:2000; #4511; Cell Signaling), mouse monoclonal anti-phospho-MAPK8/9 (Thr183/Tyr185; 1:2000; #9255; Cell Signaling), rabbit monoclonal anti-phospho-IRAK4 (Thr345/Ser346; 1:2000; #11,927; Cell Signaling), rabbit monoclonal anti-IL6 (1:2000; #12,153; Cell Signaling), TLR4 Proteintech 1:1000, and TLR4 Novus 1:1000. The membranes were incubated in the appropriate horseradish peroxidase-conjugated secondary antibody (goat anti-rabbit; 1:2000; #7074; Cell Signaling or rabbit anti-mouse; 1:2000; #7076; Cell Signaling) for 1 h at room temperature. Immunoreactive bands were visualized by chemiluminescence with an ECL kit (Thermo Fisher Scientific) in an ECL ChemoStar Imager (Intas Science Imaging, Göttingen, Germany). After detection, membranes were stripped (Elution buffer: 2% SDS, 62.5 mM Tris/HCl, pH 6.7 and 100 mM β-mercaptoethanol for 40 min at 70 °C) and incubated with the following primary antibodies: rabbit polyclonal anti-MAPK1/2 (1:2000; #9102; Cell Signaling), rabbit monoclonal anti-IKBA (1:2000; #4812; Cell Signaling), rabbit monoclonal anti-MAPK14 (1:2000; #8690; Cell Signaling), rabbit polyclonal anti-MAPK8/9 (1:2000; #9252; Cell Signaling), rabbit polyclonal anti-IRAK4 (1:2000; #4363; Cell Signaling), rabbit monoclonal anti-NFKB (1:2000; #8242; Cell Signaling), and rabbit polyclonal anti-GAPDH (1:5000; #2118; Cell Signaling). Full length recombinant protein of TLR4 (TLR4-40689H Biomart, Frankfurt, Germany) and TLR9 (TLR9-0183H Biomart) was used as a positive control.

### RNA extraction and qRT-PCR

RNA was extracted from P-IEC cells using TRIzol® (Invitrogen) and 3 µg RNA were reverse-transcribed in a final volume of 30 µL using RevertAid reverse transcriptase (Thermo Fisher Scientific) according to the manufacturer’s instructions, as previously described [21]. Briefly, quantitative mRNA expression analysis was performed by quantitative real-time PCR (qRT-PCR) using the StepOnePlus™ Real-Time PCR System (Applied Biosystems, Waltham, USA) and the PowerUp™ SYBR® Green Master Mix (Applied Biosystems) according to the manufacturer’s instructions. The final primer concentration was 0.5 μM, the final reaction volume was 20 μl, and cycle conditions were 95 °C for 2 min followed by 40 cycles of 95°C for 15 s, 60°C for 15 s, and 72 °C for 1 min. Quantitative values were obtained from the threshold cycle (*C*_*T*_) number, at which the increase in the signal associated with the exponential growth of PCR products begins to be detected. Transcript copy numbers were normalized to *GAPDH* and *RPL30* mRNA copies. Results are expressed as fold differences in target gene expression relative to *GAPDH* or *RPL30* transcripts. The Δ*C*_*T*_ value of the sample was determined by subtracting the average *C*_*T*_ value of the target gene from the average *C*_*T*_ value of the *GAPDH* or *RPL30* gene. Data analysis of *RPL30* is not shown. For each primer pair, we performed no-template control and no-RT control assays, which produced negligible signals with *C*_*T*_ values that were greater than 35. Experiments were performed in duplicates for each sample. The following primers were used: *FW-GAPDH*: 5ʹ-TCATCAACGGGAAGCCCATCAC-3′, *REV-GAPDH*: 5ʹ-AGACTCCACGACATACTCAGCACCG-3′, *FW-TLR4*: 5′-GGTGCTGGATTTATCCAGGTGTG-3ʹ, *REV*-TLR4: 5ʹ-GCTTCTGTAAACTTGATAGTCCAG-3ʹ, *FW-TLR9*: 5ʹ-CCGTGGCAATGTCACCAGC-3ʹ, *REV-TLR9*: 5ʹ-GCAGTTCCACTTGAGGTTGAG-3ʹ, *FW-RPL30*: 5ʹ-GCAGGAAGATGGTGGCCGC-3ʹ, *REV-RPL30*: 5ʹ-GCTTGTACCCCAGGACGTACTT-3ʹ.

### Statistical analysis

Data are presented as mean ± SEM and compared by Student’s *t*-test (GraphPad Prism version 5.0 for Windows, GraphPad Software, San Diego, CA, USA), and in the case of more than two groups by analysis of variance (ANOVA, one-way or two-way ANOVA) and followed by Newman-Keuls post hoc test. Group differences were considered to be statistically significant if *P* < 0.05.

## Results

### TLR9 and TLR4 are expressed in human P-IEC

**Fig. 1:**
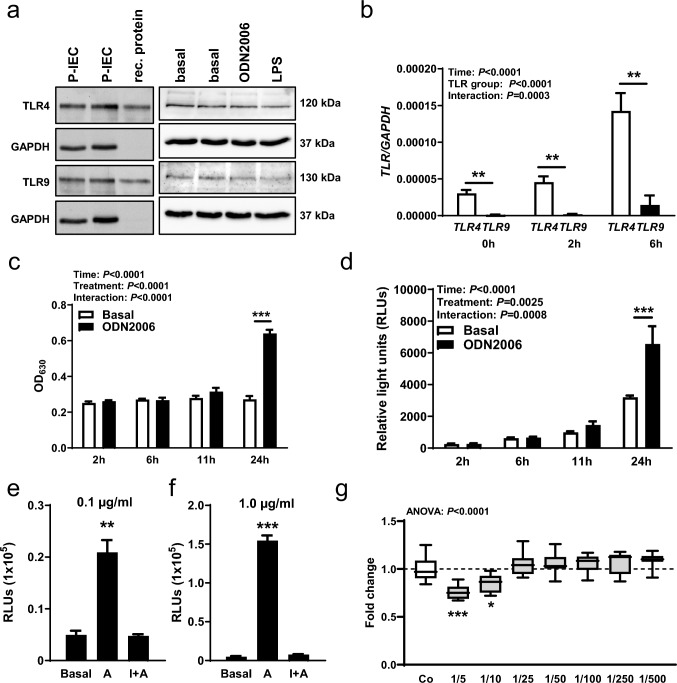
Expression and activity of TLR4 and TLR9 in human primary intestinal epithelial cells. **a** Western blot analysis of TLR4 and TLR9 in P-IECs, 0 h (basal) and 24 h after stimulation with TLR4 (LPS) or TLR9 agonist (ODN2006). GAPDH was used as reference protein. **b** Quantitative RT-PCR showing the levels of TLR4 and TLR9 in P-IECs 0 h, 2 h, and 6 h after stimulation with TLR4 (LPS) or TLR9 agonist (ODN2006). *GAPDH* was used as reference gene. n = 7/group. **c, d** HEK-Dual cells were stimulated with TLR9 agonist (ODN2006) for 2 h, 6 h, 11 h, and 24 h and the (**c**) optical density (OD_630_) or (**d**) relative light units (RLUs) were compared with non-treated cells (basal). n = 4/group. **e, f** HEK-Dual cells were treated with a TLR9 agonist (A, ODN2006) or with inhibitor (INH-18) and agonist (I + A) in two different concentrations, 0.1 µg/ml (**e**) and 1.0 µg/ml (**f**), and were compared to non-treated cells. Luminescence signal was measured with a TECAN luminescence reader. n = 4/group. **g** MTT assay showing cell viability in different dilutions of the control formula, compared to control (Co) cells incubated with medium. n = 8/group. Data were analyzed by two-way ANOVA (**b–d**) or one-way ANOVA (**g**) followed by Newman-Keuls post hoc test or Student’s *t*-test (**e, f**) and are represented as mean ± SEM. *P < 0.05, **P < 0.01, ***P < 0.001

TLRs are typically expressed in immune cells, but TLR4 and TLR9 were also detected at the protein level in human primary small intestinal epithelial cells by Western blot analysis, where recombinant full-length protein of TLR9 and TLR4 served as a positive control (Fig. 1a). Activation of TLR4 by LPS and TLR9 by ODN2006 for showed no effect on TLR protein level in intestinal epithelial cells (Fig. 1a). Expression of TLR4 and TLR9 was assessed via quantitative RT-PCR. Two and six hours after cell activation with LPS or ODN2006, respectively, the mRNA levels of TLR4 were significantly increased compared to unstimulated (0h) human P-IECs (Fig. 1b). An ANOVA showed that both receptor groups differ significantly, and that the interaction of the groups is also significantly different over time. A post hoc test showed that TLR4 is significantly more highly expressed than TLR9 at all time points (Fig. 1b).

HEK-Dual hTLR9 (NF/IL8) reporter cells were used to specifically measure TLR9 activation. Cells were stimulated with the TLR9 specific agonist ODN2006 at different time points. A significant increase of TLR9 activity was only detected by post hoc test after 24 h, compared to untreated cells (Fig. 1c, d). Analysis of IL8 quantity via QUANTI-Luc™ technology revealed an exponential increase of TLR9 activity over time, which was not confirmed by the SEAP read-out (QUANTI-Blue™). Therefore, subsequent experiments were performed with the more sensitive QUANTI-Luc™. A lack of detection of increase in TLR9 activity via QUANTI-Blue™ might be caused by cloudiness of cell medium caused by protein and fat components in IF. To analyze whether TLR9 activity could be inhibited, the TLR9 antagonist INH-18 was used prior to agonist-treatment. As demonstrated the TLR9 antagonist was able to inhibit TLR9 activity for 24 h using equivalent concentrations of 0.1 µg/ml (Fig. 1e) and 1.0 µg/ml (Fig. 1f). For all further experiments a concentration of 0.1 µg/ml was used. A MTT assay revealed reduced cell viability in IF dilutions of 1/5 and 1/10. Therefore, 1/100 dilutions were routinely used in the following experiments (Fig. 1g).

### IFs activate TLR9

**Fig. 2:**
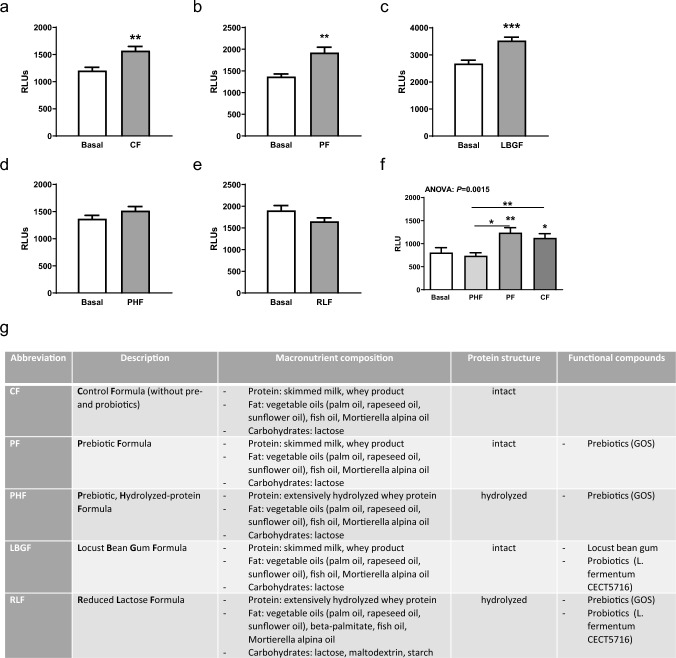
TLR9 activation by human milk or infant formulas. HEK-Dual cells were incubated for 24 h with different IF **a** CF, **b** PF, **c** LBGF, **d** PHF, and **e** RLF in a 1/100 dilution and non-treaded HEK-Dual cells served as reference. **f** Comparison of the three relevant formulas. **g** Nutritional characterization of IF. Luminescence signal was measured with a TECAN luminescence reader. n = 8/group. Data were analyzed by Student’s *t*-test (**a–e**) or one-way ANOVA (**f**) followed by Newman-Keuls post hoc test and are represented as mean ± SEM. *P < 0.05, **P < 0.01, ***P < 0.001

HEK-Dual cells were incubated with IF in a 1/100 dilution for 24 h. Control formula (CF) (Fig. 2a), infant formula containing prebiotics (PF) (Fig. 2b), and formula with locust bean gum and probiotics (LBGF) (Fig. 2c) induced a significantly higher TLR9 activation compared to non-treated cells. IF with hydrolyzed whey protein containing infant formulas PHF (Fig. 2d) and RLF (Fig. 2e) had no effect on TLR9 activation versus untreated cells. Comparing all these formulas showed the highest TLR9 activation by PF followed by CF, while PHF did not activate TLR9 signaling (Fig. 2f). Nutritional characterization of all used IFs are shown in Fig. 2g.

### Lactoferrin, L-5-methyltetrahydrofolate, and hydrolyzed whey proteins couldn’t activate TLR9

**Fig. 3:**
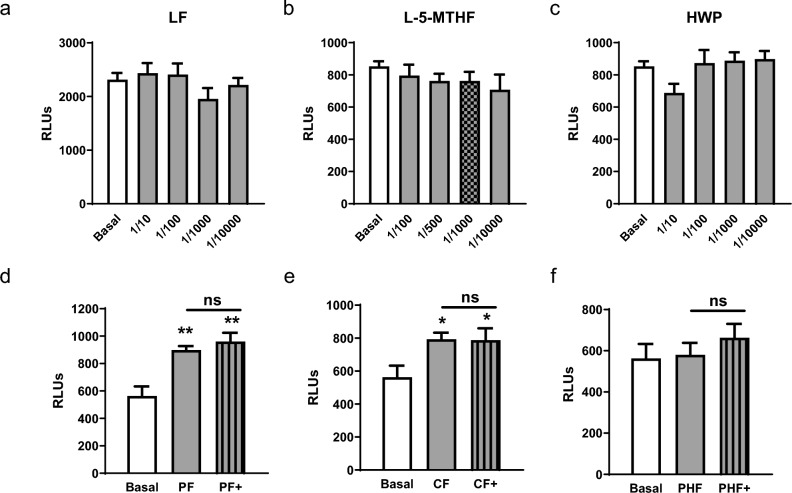
Lactoferrin, L-5-MTHF, and HWP failed to activate TLR9. **a** Lactoferrin, **b** L-5-MTHF, and **c** HWP were diluted in various concentrations from 1/10,000 up to 1/10 and applied to HEK-Dual cells. Luminescence of the supernatant was measured after 24 h and compared with luminescence of non-treated HEK-Dual cells. **d-f** Lactoferrin was added to (**d**) PF, (**e**) CF, and (**f**) PHF IF in a dilution of 1/1000 ( +). The formulas were added to the cells in a concentration of 1/100. The luminescence was measured and compared to the corresponding formula without LF and to basal (non-treated) luminescence. Luminescence signal was measured with a TECAN luminescence reader. n = 8/group. Data were analyzed by one-way ANOVA **a–c** followed by Newman-Keuls post hoc test or Student’s *t*-test **d–f** and are represented as mean ± SEM. ns = not significant, *P < 0.05, **P < 0.01

Next, TLR9 activation through the following different functional components, lactoferrin (LF), L-5-methyltetrahydrofolate (L-5-MTHF), and pure hydrolyzed whey proteins (HWP) was tested. The compounds were diluted in various concentrations from 1/10,000 up to 1/10. However, none of these compounds in any concentration were able to activate TLR9 (Fig. 3a-c). To investigate whether TLR9 activation of LF could be mediated through infant formulas, LF was added to different IFs matrices in a concentration of 1/100. LF in PF + , CF + , and PHF + showed no increase in TLR9 activity compared to formulas without LF (Fig. 3d–f). However, as already shown in the previous experiments, PF and CF (Fig. 2a, b) were able to activate TLR9, while PHF (Fig. 2d) was unfortunately unable to activate TLR9. Lactoferrin therefore appears to have no influence on TLR9 activation.

### Probiotic *L. fermentum* CECT5716 activates TLR9 signaling

**Fig. 4:**
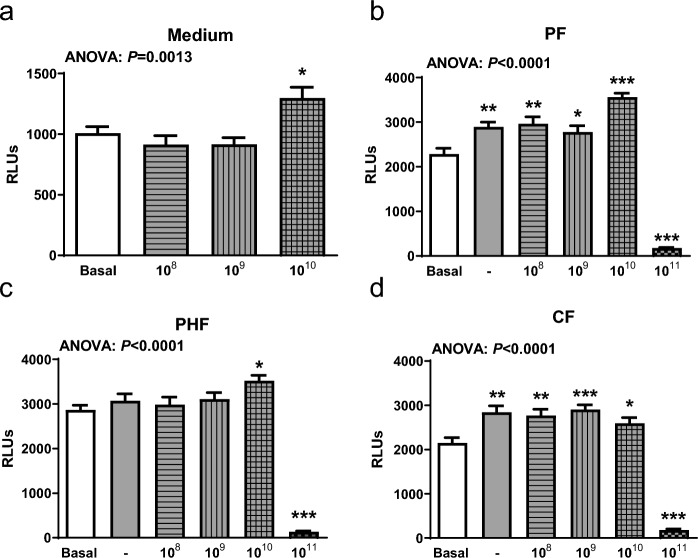
*L. fermentum* CECT5716 activates TLR9. **a** *L. fermentum* CECT5716 was added to HEK-Dual cells in various concentrations and cultivated for 24 h prior to luminescence measurement. RLUs were compared with basal HEK-Dual cells. As positive control, 0.1 µg/ml TLR9 agonist (ODN2006) was added to HEK-Dual cells. **b** PF, **c** PHF, and **d** CF formulas were added to HEK-Dual cells in a 1/100 dilution and supplemented with various concentrations of *L. fermentum* CECT5716. All results were compared to non-treated basal HEK-Dual cells and HEK-Dual cells with the corresponding infant formula in a 1/100 dilution. The luminescence signal was measured with a TECAN luminescence reader 24 h after incubation. n = 8/group. Data were analyzed by one-way ANOVA followed by Newman-Keuls post hoc test and are represented as mean ± SEM. *P < 0.05, **P < 0.01, ***P < 0.001

Since probiotic mechanisms are known to be strain specific, and TLR9 activation has been described for several probiotic strains before, we analyzed whether *L. fermentum* CECT5716 activates TLR9 signaling. Viable *L. fermentum* CECT5716 (Fig. 4a) was added to HEK-Dual cells for 24 h in concentrations of 10^8^, 10^9^, and 10^10^ CFU/mL. *L. fermentum* CECT5716 was able to activate TLR9 in a dose dependent manner. When used without IF, only high concentrations of 10^10^ CFU/mL induced TLR9 activation. To investigate, if *L. fermentum* CECT5716 could activate TLR9 in the presence of IF, *L. fermentum* CECT5716 was added at various concentrations to three different IFs to the HEK-Dual cells to measure TLR9 activity. Confirming previous results, it was clearly demonstrated that PF and CF, but not PHF, could activate TLR9 independent of *L. fermentum* CECT5716 (Fig. 4b–d). With the addition of the probiotic in high concentrations of 10^10^ CFU/mL, an additional TLR9 activation was observed in PF and PHF, but not CF. A concentration of 10^11^ CFU/mL *L. fermentum* CECT5716 resulted in significantly reduced cell viability in all used IFs shown by post hoc test (Fig. 4b–d).

### TLR9 activation level in vitro is dependent on *L. fermentum* CECT5716 viability

**Fig. 5:**
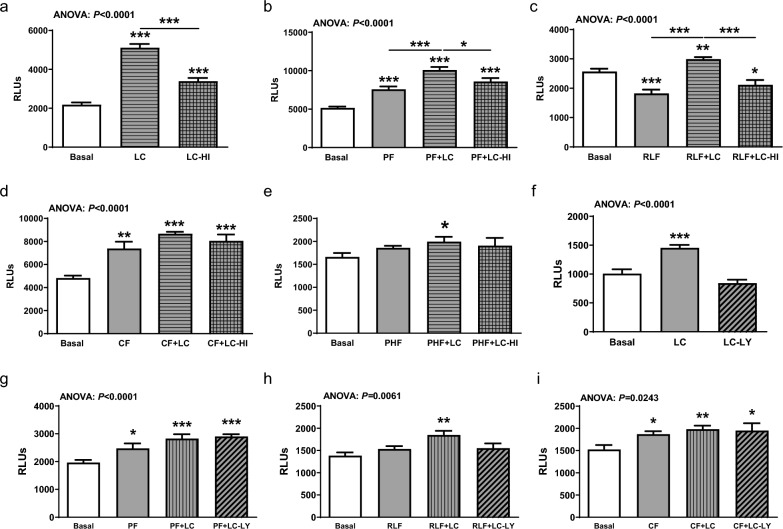
Heat-inactivated *L. fermentum* CECT5716 and lysed *L. fermentum* CECT5716 were not able to activate TLR9. **a–e** Heat-inactivated (HI) *L. fermentum* CECT5716 (LC), **f–i** lysed (LY) *L. fermentum* CECT5716, and viable *L. fermentum* CECT5716 were added to HEK-Dual cells in a concentration of 10.^10^ CFUs for 24 h before luminescence was measured and compared with basal HEK-Dual cells. PF, RLF, CF, and PHF were added to HEK-Dual cells in a 1/100 dilution. All results were compared to non-treated basal HEK-Dual cells and HEK-Dual cells with the corresponding infant formula in a 1/100 dilution. Luminescence signal was measured with a TECAN luminescence reader 24 h after incubation. n = 8/group. Data were analyzed by one-way ANOVA followed by Newman-Keuls post hoc test and are represented as mean ± SEM. *P < 0.05, **P < 0.01, ***P < 0.001

HEK-Dual cells incubated for 24 h with viable or heat-inactivated *L. fermentum* CECT5716 showed a significant increase of TLR9 activation compared to non-treated HEK-Dual cells (Fig. 5a). Interestingly, viable *L. fermentum* CECT5716 induced significantly higher TLR9 activation compared to heat-inactivated *L. fermentum* CECT5716 (Fig. 5a). This pattern was also observed in PF and RLF, but not in CF (Fig. 5b–d). While PHF again showed no activation of TLR9 signaling, the addition of viable but not heat-inactivated *L. fermentum* CECT5716, induced a slight increase in TLR activation (Fig. 5e).

As heat inactivation of *L. fermentum* CECT5716 might destroy essential structural components necessary for TLR9 activation, *L. fermentum* CECT5716 inactivated by mechanical lysis was also tested for its TLR9 stimulating potential. Similar results were observed when *L. fermentum* CECT5716 was lysed and incubated with different IFs (Fig. 5f–i). Lysed *L. fermentum* CECT5716 in medium showed no significant TLR9 activation compared to viable *L. fermentum* CECT5716 in medium (Fig. 5f). For PF and CF we only detected TLR9 activation mediated by formula. While RLF showed no TLR9 activation, the addition of viable *L. fermentum* CECT5716 resulted in a TLR9 activating effect in contrast to RLF with lysed *L. fermentum* CECT5716 (Fig. 5g–i).

### Human P-IECs show decreased IL6 levels when incubated with IFs and vice versa TLR9 inhibition increased IL6 levels

**Fig. 6:**
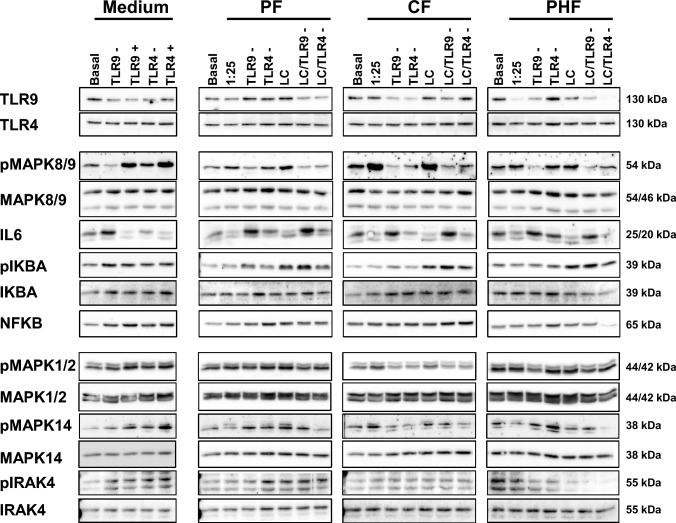
IL6 levels were decreased through various infant formulas in human primary intestinal epithelial cells; in contrast IL6 levels were increased due to TLR9 inhibition. The following phosphorylation stages and proteins were investigated by Western blot analysis in human P-IECs: TLR9, TLR4, pMAPK1/2 and MAPK1/2, pMAPK8/9 and MAPK8/9, pp38 and p38, pIRAK4 and IRAK4, IL6, NFKB, pIKB and IKB. HEK-Dual cells were cultivated in medium, PF, CF, and PHF in a 1/25 dilution, with TLR9 and TLR4 inhibitor (0.1 µg/ml) and non-treated HEK-Dual cells served as reference. In addition, the cells were incubated with *L. fermentum* CECT5716 (LC) (10^10^ CFUs) for 30 min. GAPDH was used as reference protein

Based on our previous results, we treated human P-IECs with different IFs to investigate levels of downstream signaling proteins of TLR9 and TLR4. To evaluate TLR9- and TLR4-specific effects in P-IECs, cells were additionally inhibited with a TLR9 or TLR4 antagonist prior to stimulation. IFs did not influence TLR4 and TLR9 protein levels in P-IECs. As expected, after inhibition of TLR9, interleukin (IL6) levels were highly increased. This effect was independent of *L. fermentum* CECT5716 treatment (Fig. 6). IL6 levels were decreased in the presence of all IFs compared to non-treated P-IECs. In combination with inhibition of TLR9, dramatically increased IL6 levels were observed (Fig. 6). Inhibition of TLR4 did not alter the amount of IL6 in P-IECs. The IL6 antibody can detect two human isoforms of IL6, but only the larger isoform plays an important role in inflammatory responses. Mitogen-activated protein kinase 8/9 (MAPK8/9) showed reduced phosphorylation when P-IECs were inhibited with TLR9 antagonist and in contrast highly phosphorylated when incubated with IF (Fig. 6). In P-IECs incubated with PHF, phosphorylation levels of MAPK8/9 were only slightly increased (Fig. 6).

In P-IECs, *L. fermentum* CECT5716 can activate NF-kappa-B inhibitor alpha (IKBA) through serine 32 phosphorylation, but independent of TLR4 and TLR9 activity (Fig. 6). Phosphorylation of IKBA results in the translocation of the transcription factor nuclear factor kappa B (NFKB) from the cytosol into the nucleus. Several kinases such as mitogen-activated protein kinase 1/2 (MAPK1/2), mitogen-activated protein kinase 14 (MAPK14), and interleukin-1 receptor-associated kinase 4 (IRAK4), which are downstream of the TLR9 signaling pathway, showed no alteration neither with infant formula nor with any TLR inhibitor (Fig. 6).

## Discussion

In this study, we tested whether various infant formulas and the probiotic *Limosilactobacillus fermentum* CECT5716 could activate TLR9 to stimulate the gastrointestinal anti-inflammatory machinery. We used genetically designed HEK-Dual™ hTLR9 (NF/IL8) cells, overexpressing human TLR9 with a triple knockout of endogenous TLR3, TLR5, and TNFR to avoid unspecific activation. Finally, we validated the findings in P-IECs and analyzed TLR downstream signaling as well as IL6 expression.

TLR4 and TLR9 are involved in inflammatory processes and seem to play an important role in inflammatory diseases such as NEC [15, 22–24]. TLR4 is strongly activated during inflammatory diseases of the intestine in preterm infants [15, 25, 26]. Interestingly, TLR4 is highly expressed in intestinal epithelial cells of infants during the end of gestation, shortly before the microbiome colonizes the gut after birth. Contrary, and at the same time, expression of anti-inflammatory TLR9 receptor is very low [11]. As a result, the relation of TLR4/TLR9 expression in epithelial intestine cells is changed after birth. It seems that TLR4 and TLR9 complement and mutually influence each other during inflammation, but their functions in the gut are not yet fully understood [13, 15, 25, 27]. Human milk supports infants during NEC by activating and stabilizing TLR9 instead of TLR4 and shifts the intestinal epithelium toward an anti-inflammatory environment [22, 28, 29]. In our current study, we investigated whether various IF could also activate TLR9. We demonstrated that the formulas with intact protein (CF, PF, LBGF) were able to activate TLR9, in contrast to PHF and RLF, which both contained hydrolyzed whey proteins. We speculate that small peptides, generated during the hydrolysis process, prevent TLR9 activation and could possibly act as TLR9 inhibitors. However, we were not able to identify any compound in the formulas which is responsible for TLR9 activation. We investigated lactoferrin and L-5-MTHF [30], as well as hydrolyzed whey protein, but none of these compounds were able to activate TLR9. Interestingly, a NEC preventing effect of lactoferrin was found in preterm infants [31, 32] possibly by acting through a non-TLR9 mediated mechanism. These results are not surprising, due to the fact that TLR9 could be activated by CpG-DNA fragments [14, 18].

Probiotics such as lactobacilli could serve as a source for CpG-DNA, but CF and PF are free of any probiotics, and both are able to activate TLR9 [26]. Therefore, we speculate that other compounds in IF are also able to activate TLR9 by a non-CpG mediated mechanism. In literature it was shown that different gram-positive lactobacilli, e.g. *Lactobacillus rhamnosus* HN001 [33] are able to activate TLR9 and improve enterocolitis in neonatal mice or preterm piglets [19]. Furthermore, it was demonstrated that probiotic strains can reduce the incidence of NEC and support intestinal epithelial homeostasis [34–40]. In our study we demonstrated that *L. fermentum* CECT5716 was also able to activate TLR9 at high concentrations. Previously, it was reported that *L. fermentum* CECT5716 has anti-obesogenic and anti-inflammatory effects [41]. When viable *L. fermentum* CECT5716 was added to PF, TLR9 activation was further increased. This effect could also be measured in PHF upon addition of *L. fermentum* CECT5716. The reason for the increased TLR9 activation following the addition of *L. fermentum* CECT5716 could be the presence of prebiotics that are contained in PF and PHF formulas indicating a synergistic effect of *L. fermentum* CECT5716 in combination with GOS. CF formula lacks GOS and showed no increase in TLR9 activity after *L. fermentum* CECT5716 supplementation. Overall, *L. fermentum* CECT5716 seems to be a suitable TLR9 activator and could therefore serve as an additive in formulas without TLR9 activating effect in the future. Interestingly, only viable *L. fermentum* CECT5716 has an activating effect on TLR9. When *L. fermentum* CECT5716 was heat-inactivated, no increase in TLR9 activity was observed. A similar picture emerged when *L. fermentum* CECT5716 was inactivated by cell lysis. Lysed *L. fermentum* CECT5716 showed no activation of TLR9 and when added to CF, PF, RLF, or PHF we only measured the basal formula effect on TLR9 activation. Good et al. 2014 demonstrated that *Lacticaseibacillus rhamnosus* HN001, which was inactivated via UV light irradiation, could still improve enterocolitis in preclinical studies, when administered to mice and piglets [19]. In contrast to heat inactivation and lysis, UV light inactivation is a mild inactivation process and the bacteria remain viable compared to the previously discussed methods. However, since heat inactivation and cell lysis seem to inhibit TLR9 activation of *L. fermentum* CECT5716 in intestinal epithelial cells, supplementation of living *L. fermentum* CECT5716 is favored.

In addition, we investigated the downstream signaling pathways of TLR4 and TLR9 receptors in P-IECs and detected no differences in phosphorylation levels of the most important kinases of the TLRs, IRAK4 and MAPK1/2. However, both TLRs bind MYD88 and build a complex together with IRAK4, IRAK1, IRAK2, and TRAF6 after receptor activation, therefore it is difficult to assign changes in the signaling pathway to a specific TLR receptor, when both receptors are activated at the same time [7]. Therefore, to prevent concomitant activation of TLR4 and TLR9, we used TLR4 and TLR9 inhibitors, to distinguish between TLR4 and TLR9 activation and signaling, respectively. We clearly detected decreased phosphorylation levels of MAPK8/9 in P-IECs after TLR9 inhibition and simultaneously increased levels of IL6. Contrary, we found increased levels of phosphorylated MAPK8/9 and decreased levels of IL6 in P-IECs cultured with CF and PF compared with non-treated cells. MAPK proteins are downstream signaling kinases of the TLR pathways and activate different transcription factors which could change the cell behavior after TLR activation in a proinflammatory manner [42]. However, it was shown that the inhibition of MAPK8/9, also known as stress activated protein kinase (SAPK), reduces the toxic effects of LPS in the RAW 264.7 macrophage cell line, as also shown for TLR4 and NFKB inhibition [43]. IL6 is secreted by many epithelial and endothelial cells. Besides IL1B and tumor necrosis factor alpha, IL6 is one of the early interleukins and key regulators during inflammation and increased IL6 levels are associated with an acute inflammatory process [44].

We are aware that our study has one major limitation, as it is a fully in vitro study and we have not tested the used IFs and the positive effect of TLR9 activation in vivo. The IFs have also not been digested, as they would have been under in vivo circumstances, and thus might have a different effect on the intestinal cells and on tissue damaged by NEC. These investigations should definitely be carried out in a further in vivo study. However, our data reveal that TLR9 activity induced by different IFs or *L. fermentum* CECT5716 results in decreased IL6 expression in vitro. Furthermore, we detected that *L. fermentum* CECT5716 phosphorylates IKBA, a signaling molecule which ensures that the transcription factor NFKB transfers into the nucleus after IKBA phosphorylation.

**Fig. 7:**
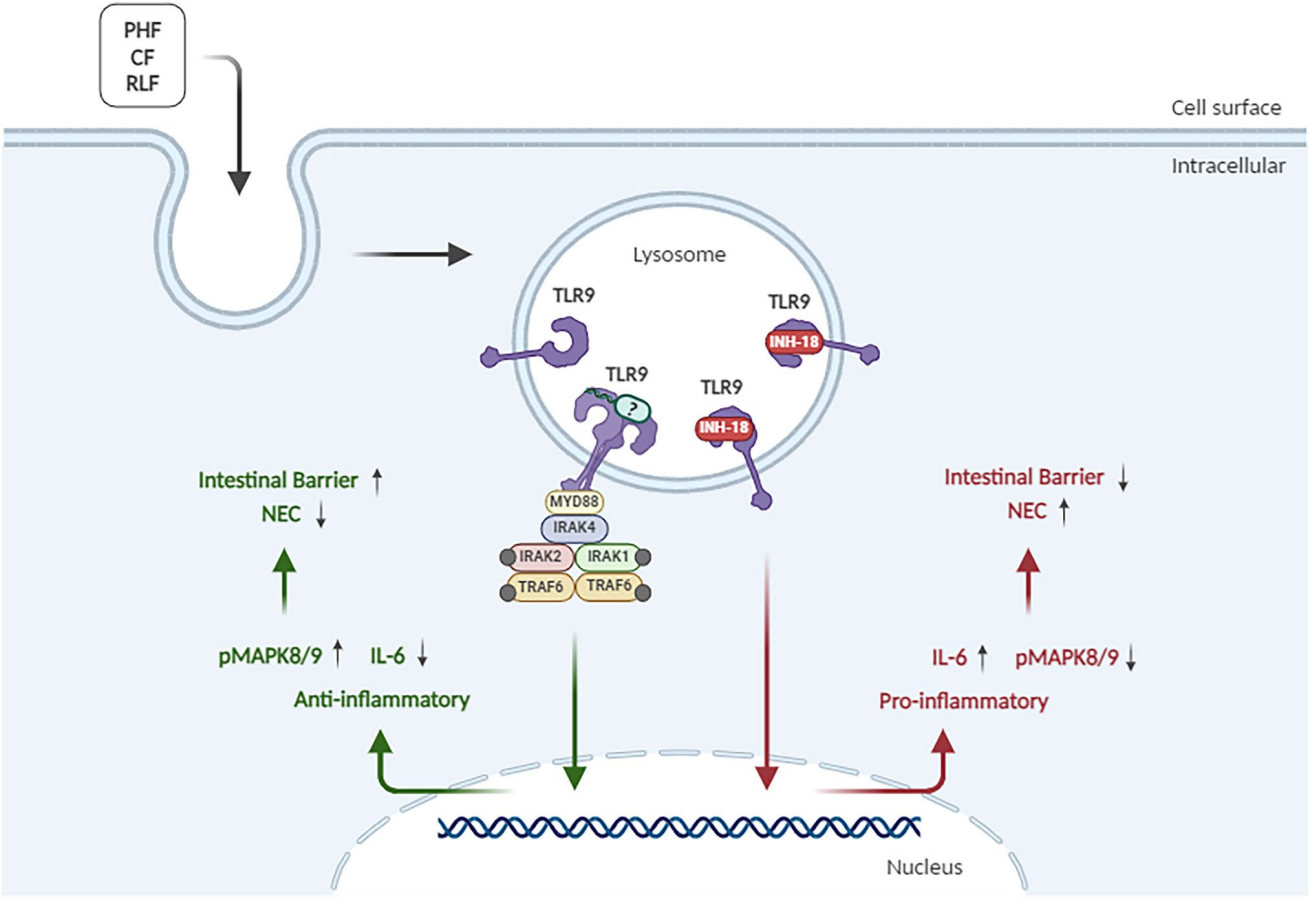
Scheme summarizing the results of our analyses. The image was created by using the BioRender software (BioRender, Toronto, Canada)

In conclusion, our study showed that PF, LBGF, CF, and *L. fermentum* CECT5716 can activate TLR9 in human P-IECs and reduce IL6 levels, what could have an anti-inflammatory effect in NEC and other bowel diseases (Fig. 7). Since it is known that TLR9 activity could reduce the inflammatory process during NEC, *L. fermentum* CECT5716 and the relevant IF could be a promising approach for infants with a risk for NEC who cannot be breastfed.

## Data Availability

Data will be made available on request.
